# Boosting High‐Rate Li–S Batteries by an MOF‐Derived Catalytic Electrode with a Layer‐by‐Layer Structure

**DOI:** 10.1002/advs.201802362

**Published:** 2019-07-15

**Authors:** Wanlong Li, Ji Qian, Teng Zhao, Yusheng Ye, Yi Xing, Yongxin Huang, Lei Wei, Nanxiang Zhang, Nan Chen, Li Li, Feng Wu, Renjie Chen

**Affiliations:** ^1^ Beijing Key Laboratory of Environmental Science and Engineering School of Material Science and Engineering Beijing Institute of Technology Beijing 100081 P. R. China; ^2^ Collaborative Innovation Center of Electric Vehicles in Beijing Beijing 100081 P. R. China

**Keywords:** catalytic effect, layer‐by‐layer, lithium–sulfur batteries, MOF‐derived, ultrafine CoS_2_ nanoparticles

## Abstract

Rechargeable high‐energy lithium–sulfur batteries suffer from rapid capacity decay and poor rate capability due to intrinsically intermediate polysulfides' shuttle effect and sluggish redox kinetics. To tackle these problems simultaneously, a layer‐by‐layer electrode structure is designed, each layer of which consists of ultrafine CoS_2_‐nanoparticle‐embedded porous carbon evenly grown on both sides of reduced graphene oxide (rGO). The CoS_2_ nanoparticles derived from metal–organic frameworks (MOFs) have an average size of ≈10 nm and can facilitate the conversion between Li_2_S_6_ and Li_2_S_2_/Li_2_S in the liquid electrolyte by a catalytic effect, leading to improved polysulfide redox kinetics. In addition, the interconnected conductive frameworks with hierarchical pore structure afford fast ion and electron transport and provide sufficient space to confine polysulfides. As a result, the layer‐by‐layer electrodes exhibit good rate capabilities with 1180.7 and 700 mAh g^−1^ at 1.0 and 5.0 C, respectively, and maintain an impressive cycling stability with a low capacity decay of 0.033% per cycle within ultralong 1000 cycles at 5.0 C. Even with a high sulfur loading of 3.0 mg cm^−2^, the electrodes still show high rate performance and stable cycling stability over 300 cycles.

## 1 Introduction

Compared with the currently commercial lithium‐ion batteries, lithium–sulfur (Li–S) batteries, as the potential next‐generation energy storage device, are very attractive due to their low cost, high theoretical capacity (1675 mAh g^−1^), and high energy density (2600 Wh kg^−1^).^1^ However, the worldwide application of Li–S batteries has not been achieved because of several major obstacles: 1) poor rate performance resulting from the low electronic conductivity of sulfur and its discharge products (Li_2_S and Li_2_S_2_);^2^ 2) the rapid capacity decay caused by the dissolution and parasitic shuttle effect of the intermediate lithium polysulfides (Li_2_S*_n_*, 4 ≤ *n* ≤ 8) in organic liquid electrolyte;^3^ and 3) slow redox kinetics due to the transformation between different active materials phases.^4^

Over the past decades, many efforts have been made to advance Li–S batteries.^5^ Among them, constructing novel sulfur–carbon composite cathodes with porous and conductive carbon matrices has been proved effective. The carbon host, such as carbon nanotubes,^6^ micro/mesoporous carbons,^7^ graphene,^8^ and hollow carbon spheres,^9^ has large pore volume and excellent electrical conductivity, which can adsorb lithium polysulfides physically and improve the utilization of active materials to some extent. However, individual carbon particles are lack of interfacial connectivity and conductivity due to their separate dispersion, resulting in poor rate performance.^10^ Hence, it necessitates the design of an interconnected carbon conductive networks, which is favorable for fast electron and ion transfer.^11^ Moreover, the nonpolar carbon materials only provide weak physical confinement to polar lithium polysulfides, leading to the detachment of lithium polysulfides during long‐term cycling.^12^ For this reason, polar metal compounds are introduced to act as chemical trappers.^13^ For example, nanostructured metal oxides, such as TiO_2_,^14^ Al_2_O_3_,^15^ and MnO_2_,^16^ are efficient in entrapping lithium polysulfides via lithium–oxygen binding and metal–sulfur binding.^17^ However, these metal oxides with relatively poor electrical conductivity tend to slow down the redox kinetics of polysulfides.^18^ The sluggish redox kinetics of polysulfides is another factor that restricts rate capability by hindering the fast and full conversion between soluble polysulfides and Li_2_S_2_/Li_2_S.^19^ Electrocatalysis is considered a very effective approach to facilitate reaction kinetics.[qv: 4c,20] Recently, metal sulfides (CoS_2_,^21^ Co_9_S_8_,^22^ TiS_2_,^23^ NiS,^24^ FeS_2_,^25^ ZnS,^26^ and MoS_2_ 27) have been widely used in sulfur cathode in order to catalyze the redox kinetics of polysulfides. To further increase such function, it is required to avoid the aggregation of catalyst during the synthesis process. Thus, controlling the size of these metal sulfides becomes very critical.[qv: 12b,28] It is well known that metal–organic frameworks (MOFs) with highly controllable pore structures and multiple composition are promising templates to fabricate untrafine metal–compound nanoparticles owing to the highly dispersed metal sites inside MOFs.^29^

Inspired by this, we prepared the continuously layered‐by‐layered carbon nanosheets with embedded ultrafine CoS_2_ nanoparticles (CoS_2_‐LBLCN). Each layer of the carbon nanosheets consists of two layers of bimetallic zeolite imidazole framework (BMZIF)‐derived ultrafine CoS_2_ nanoparticles embedded porous carbon densely grown on both sides of reduced graphene oxide (rGO). The BMZIF‐derived untrafine CoS_2_ nanoparticles not only provide sufficient contact active sites for strong chemical adsorption of polysulfides, but also further accelerate the polysulfides redox kinetics by catalytic effect, thus improving the rate capability and cycling stability. In addition, the interconnected conductive framework with hierarchal pore structure can facilitate rapid ion and electron transport and provide sufficient space to restrict the diffusion of soluble polysulfides. With these synergistic advantages, the CoS_2_‐LBLCN‐based sulfur cathodes deliver a superior cycling stability with a capacity decay of 0.033% per cycle over 1000 cycles and good rate capabilities with 1180.7 and 700 mAh g^−1^ at 1.0 and 5.0 C, respectively. Even with a high sulfur loading of 3.0 mg cm^−2^, the cathodes still show high specific capacity and stable cycle stability over 300 cycles at high current rates.

## 2 Results and Discussion

**Figure 1:**
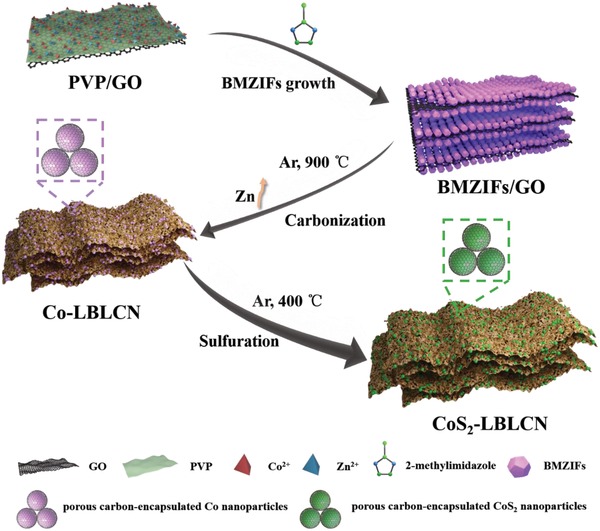
Schematic illustration of the synthesis process of CoS_2_‐LBLCN.

The synthesis process of CoS_2_‐LBLCN is illustrated in **Figure** **1**. First, graphene oxide (GO) was dispersed homogeneously in methanol solution by ultrasonication, followed by poly(vinylpyrrolidone) (PVP) modification to enrich its surface functional groups. Zn(NO_3_)_2_·6H_2_O and Co(NO_3_)_2_·6H_2_O with a molar rate of 2:1 were then added to the above solution. The Zn^2+^ and Co^2+^ were absorbed on the modified GO surface through the coordination interaction with the amide carbonyl groups of PVP.^30^ After the addition of 2‐methylimidazole solution, BMZIFs were grown uniformly on both sides of GO nanosheets (BMZIFs/GO). The obtained dark purple BMZIFs/GO powder (Figure S1, Supporting Information) was subsequently carbonized at 900 °C to produce Co nanoparticles embedded layer‐by‐layer carbon nanosheets (Co‐LBLCN) under Ar. During carbonization, the Zn‐containing components were reduced by carbon and evaporated, resulting in a hierarchical micro‐mesoporous structure.^31^ After that, the sulfuration process was conducted in a tube furnace at 400 °C, during which the remained Co nanoparticles in Co‐LBLCN reacted with sublimed sulfur to form ultrafine CoS_2_ nanoparticles. Finally, the black CoS_2_ nanoparticles embedded layer‐by‐layer carbon nanosheets (CoS_2_‐LBLCN) powder were produced (Figure S1, Supporting Information). As the control sample, the layer‐by‐layer carbon nanosheets (LBLCNs) without embedded CoS_2_ nanoparticles were also synthesized (Figure S2, Supporting Information).

**Figure 2:**
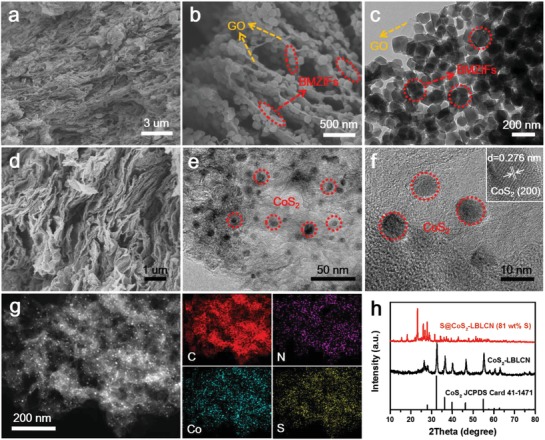
a,b) SEM and c) TEM images of the BMZIFs/GO. d) SEM and e,f) TEM images of CoS_2_‐LBLCN. g) TEM image of CoS_2_‐LBLCN and corresponding EDX elemental mapping images of C, N, Co, and S. h) XRD patterns of CoS_2_‐LBLCN and S@CoS_2_‐LBLCN.

The morphology and microstructures of BMZIFs/GO and CoS_2_‐LBLCN were investigated by scanning electron microscopy (SEM) and transmission electron microscopy (TEM). From low‐magnification SEM image in **Figure** **2**a, it can be clearly observed that the BMZIF nanoparticles are grown densely on both sides of the GO to form a layer‐by‐layer 2D nanosheet. The SEM image in Figure 2b and the TEM image in Figure 2c further confirm the assembly of GO and BMZIFs having an average size of 100 nm. Interestingly, the assembled 2D nanosheets can stack into a 3D macrostructure, as shown in Figure 2a,b. Figure 2d,e shows the SEM and TEM images of the CoS_2_‐LBLCN. After carbonization and sulfuration treatments, the 2D nanosheets shrink slightly, and BMZIF‐derived porous carbons are covered on the rGO such that the continuous conductive carbon networks are formed. In addition, the well‐stacked 3D macrostructure of the BMZIFs/GO still remained, which can provide sufficient space for sulfur incorporation. Further from the TEM images in Figure 2e,f, it can be clearly seen that the ultrafine CoS_2_ nanoparticles with an average diameter of 10 nm are uniformly embedded in the carbon matrix. The high‐resolution TEM (HRTEM) image (Figure 2f, inset) also shows obvious parallel lattice fringes with a spacing of 0.276 nm for the (200) plane of CoS_2_. Figure 2g presents the energy‐dispersive X‐ray spectroscopy (EDX) elemental mapping of the CoS_2_‐LBLCN, which exhibits the uniform distribution of elemental C, N, Co, and S in the CoS_2_‐LBLCN composite. X‐ray photoelectron spectroscopy (XPS) analysis further confirms the existence of C, N, Co, and S (Figure S4, Supporting Information). The crystallinity of the as‐prepared CoS_2_‐LBLCN and S@CoS_2_‐LBLCN was confirmed by X‐ray diffraction (XRD) (Figure 2h). The broad diffraction peak around 26° is assigned to the (002) plane of carbon from rGO and BMZIF‐derived porous carbon. The remaining peaks at 32.3°, 36.2°, and 54.9° match well with the characteristic peaks of standard CoS_2_ (JCPDS Card No. 41–1471). The broadening of these peaks shows the ultrafine features of the as‐prepared CoS_2_ nanoparticles.^32^ After introducing sulfur, the characteristic peaks of F*ddd* orthorhombic sulfur appear in the XRD pattern of S@CoS_2_‐LBLCN composite. The morphology of S@CoS_2_‐LBLCN is shown in Figure S5 (Supporting Information), which is similar to that of CoS_2_‐LBLCN. No agglomeration of sulfur is observed at the surface of the S@CoS_2_‐LBLCN, which is benefited from the large specific area (520 m^2^ g^−1^) and hierarchal pore structure of CoS_2_‐LBLCN (Figure S6, Supporting Information). The sulfur content of the S@CoS_2_‐LBLCN composite is 81 wt% measured by thermal gravity analysis (TGA) under N_2_ (Figure S7, Supporting Information).

To evaluate the effect of the CoS_2_ nanoparticles on the performance of Li–S batteries, coin cells with @CoS_2_‐LBLCN and S@LBLCN cathodes were assembled. Figure S8a (Supporting Information) shows the cyclic voltammetry (CV) curves of the S@CoS_2_‐LBLCN cathode for the initial five cycles at a scan rate of 0.1 mV s^−1^. During the cathodic scan, the two peaks at 2.36 and 2.03 V can be attributed to the reduction of S_8_ to Li_2_S*_n_* (4 ≤ *n* ≤ 8) and the subsequent conversion of Li_2_S*_n_* (4 ≤ *n* ≤ 8) to Li_2_S_2_/Li_2_S, respectively. In the anodic scan, two peaks at 2.32 and 2.43 V correspond to the oxidation of Li_2_S_2_/Li_2_S to Li_2_S*_n_* (4 ≤ *n* ≤ 8), and Li_2_S*_n_* (4 ≤ *n* ≤ 8) ultimately transforms to S_8_. Compared to S@CoS_2_‐LBLCN cathode, S@LBLCN cathode only exhibits one broad oxidation peak, whose position shifts from 2.32 to 2.42 V (Figure S8b, Supporting Information). In addition, its reduction peak shifts from 2.03 to 1.99 V. These results confirm that CoS_2_ nanoparticles can catalyze the redox reaction of polysulfides, leading to improved redox reversibility. After that, the scan rate was further increased from 0.1 to 2 mV s^−1^ (Figures S9 and S10, Supporting Information). The S@CoS_2_‐LBLCN cathode shows much better reversibility and redox kinetics at high scan rates. The specific redox peaks at different scan rates are shown in Figure S11 (Supporting Information).

**Figure 3:**
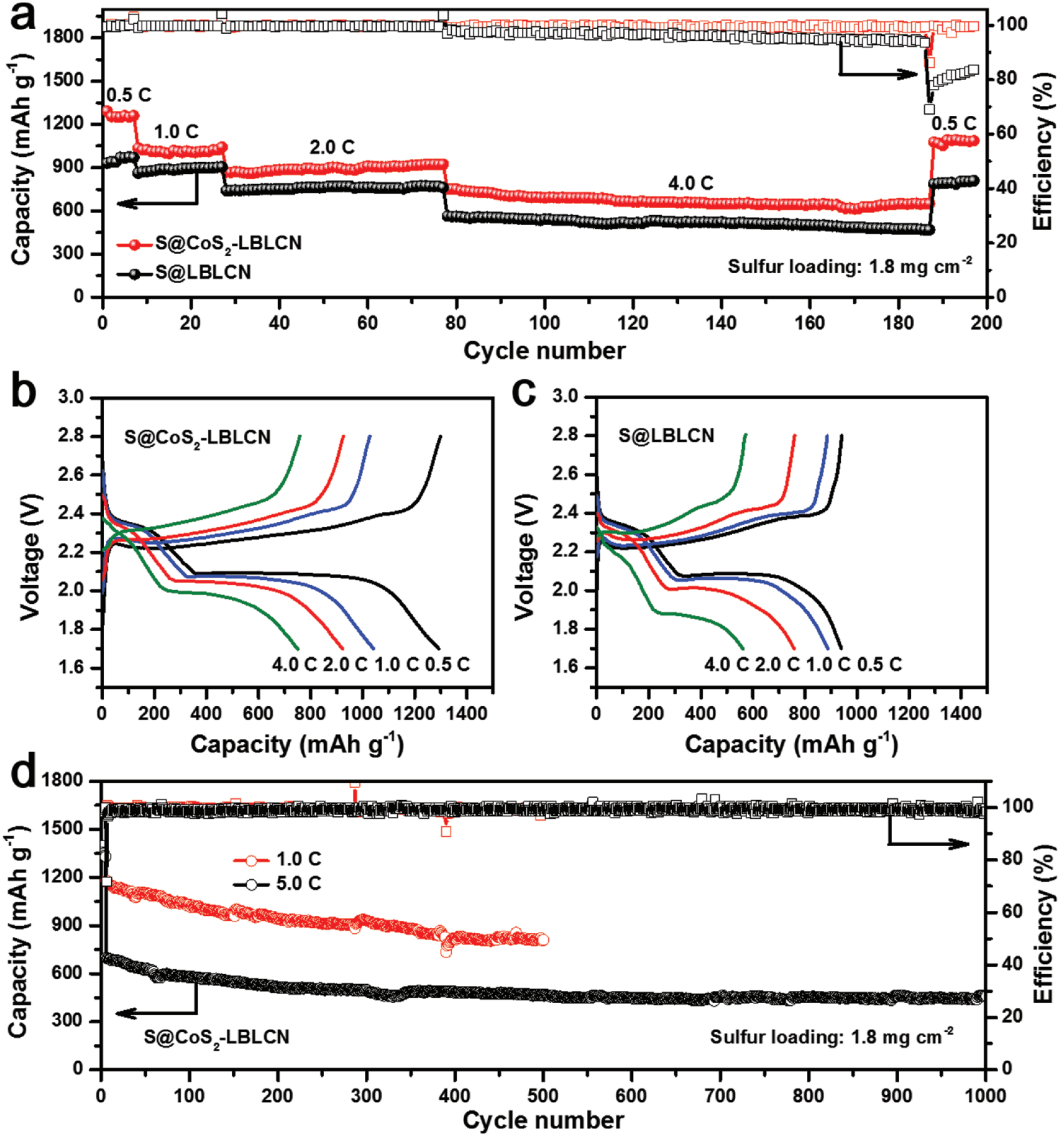
a) Rate performances of S@CoS_2_‐LBLCN and S@LBLCN cathodes at different current rates. b,c) Charge/discharge profiles of S@CoS_2_‐LBLCN (b) and S@LBLCN (c) cathodes at different rates. d) Cycling performances of S@CoS_2_‐LBLCN cathodes at high current rates of 1.0 and 5.0 C.

The cycling performances of S@CoS_2_‐LBLCN and S@LBLCN cathodes at 0.2 C are plotted in Figure S12 (Supporting Information). The S@CoS_2_‐LBLCN cathode delivers a high initial discharge capacity of 1435.8 mAh g^−1^ and maintains a reversible capacity of 1225.9 mAh g^−1^ after 50 cycles with a Coulombic efficiency of nearly 100%. In contrast, the S@LBLCN cathodes have an initial reversible capacity of 1315.9 mAh g^−1^ and preserve the discharge capacity of 1030.6 mAh g^−1^ after 50 cycles. **Figure** **3**a depicts the rate performances of the S@CoS_2_‐LBLCN and S@LBLCN cathodes at different current rates from 0.5 to 4.0 C. The S@CoS_2_‐LBLCN cathode delivers a stable discharge capacity of 1259.6 mAh g^−1^ at the seventh cycle of 0.5 C, which is around 29.8% higher than that of the S@LBLCN cathode. When cycled stepwise at the current rates of 1.0, 2.0, and 4.0 C, the reversible capacity of S@CoS_2_‐LBLCN cathode can maintain at 1042.1, 923.3, and 753.1 mAh g^−1^, respectively (Figure 3a). In contrast, the S@LBLCN cathode shows much poor capacities under the same conditions. When the current rate returned to 0.5 C, a stable discharge capacity of 1095 mAh g^−1^ is recovered for the S@CoS_2_‐LBLCN cathode, which is about 35.8% higher than the reference. These results confirm that S@CoS_2_‐LBLCN cathodes have better rate tolerance, which is attributed to its lower charge transfer resistance (Figure S13, Supporting Information). Meanwhile, the S@CoS_2_‐LBLCN cathode has much higher Coulombic efficiency than S@LBLCN cathode, especially at high rate. Figure 3b,c depicts the galvanostatic charge/discharge profiles of S@CoS_2_‐LBLCN and S@LBLCN cathodes at different current rates within the voltage window of 1.7–2.8 V. Although the discharge profiles of both S@CoS_2_‐LBLCN and S@LBLCN cathodes exhibit a distinct two‐plateau behavior at all current rates, smaller potential polarization is observed for S@CoS_2_‐LBLCN cathode with a value of 198.6, 227.5, 265.3, and 380.3 mV from 0.5 to 4.0 C (Figure S14, Supporting Information).

The long‐term cycling stability of the Li–S batteries with the S@CoS_2_‐LBLCN cathodes was further investigated at high current rates of 1.0 and 5.0 C, respectively (Figure 3d). The S@CoS_2_‐LBLCN cathode delivers an initial capacity of 1180.7 mAh g^−1^ at 1.0 C and maintains at 810.8 mAh g^−1^ after 500 cycles. When the current rate increases to 5.0 C, the initial capacity reduces to 700 mAh g^−1^, which gradually stabilizes at 463.3 mAh g^−1^ after 1000 cycles with a low capacity decay of 0.033% per cycle. In addition, the Coulombic efficiencies of the S@CoS_2_‐LBLCN cathodes over long‐term cycling at both rates are quite high, reaching around 99%.

**Figure 4:**
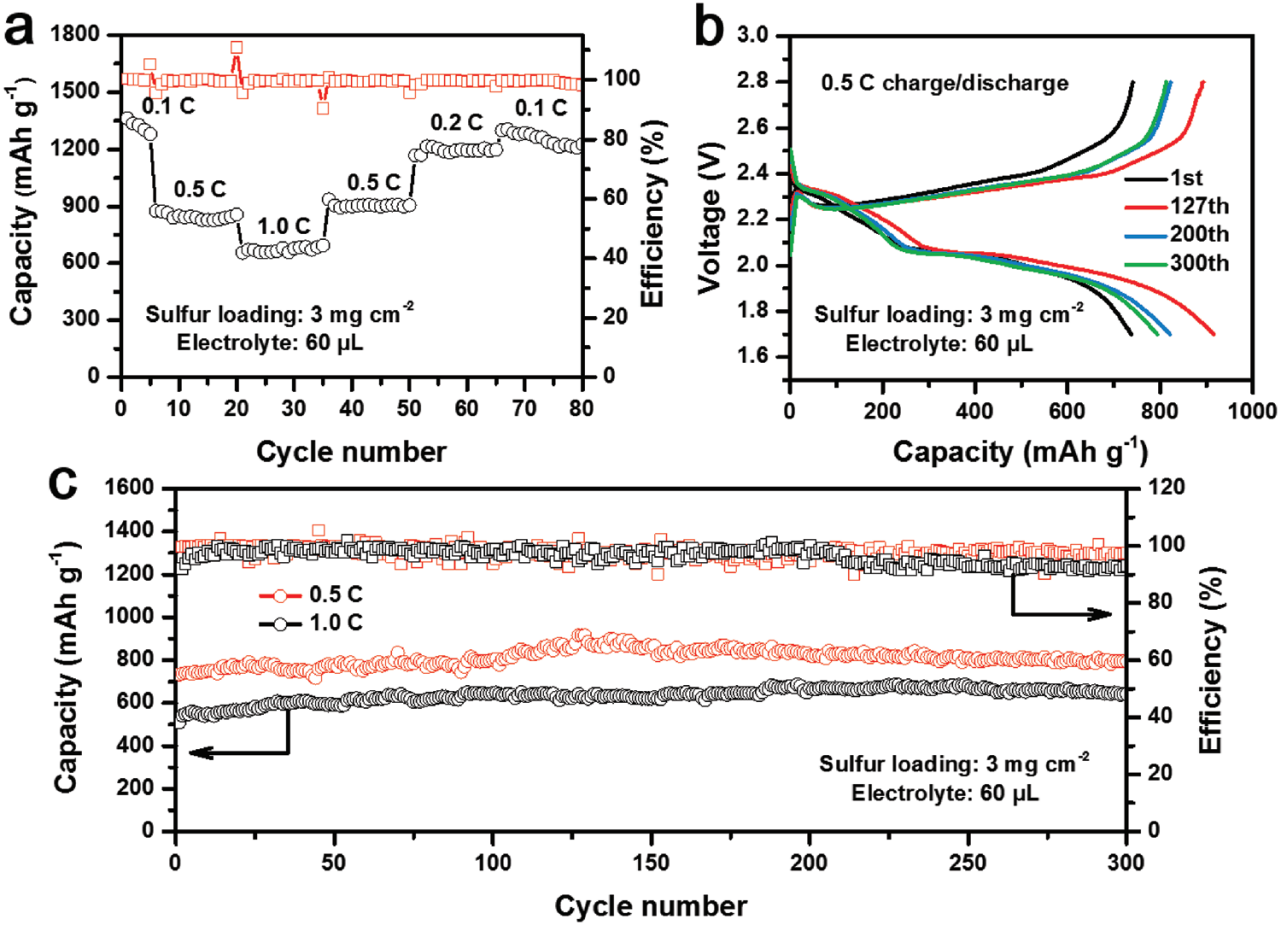
a) Rate performance of S@CoS_2_‐LBLCN cathode with a sulfur loading of 3.0 mg cm^−2^. b) Charge/discharge profiles of S@CoS_2_‐LBLCN cathode at 0.5 C. c) Cycling performances of high sulfur loading S@CoS_2_‐LBLCN cathodes at 0.5 and 1.0 C over 300 cycles.

S@CoS_2_‐LBLCN cathodes with a high sulfur loading of 3.0 mg cm^−2^ were also prepared to investigate the rate capability and cycling performance. **Figure** **4**a and Figure S15 (Supporting Information) show the rate performance of the S@CoS_2_‐LBLCN cathode at different current rates. The discharge capacities of the S@CoS_2_‐LBLCN cathode are 1363.1, 875.6, and 694.4 mAh g^−1^ at 0.1, 0.5, and 1.0 C, respectively. Reversible capacities of 905.9, 1215.6, and 1304.4 mAh g^−1^ are observed when the current rates return to 0.5, 0.2, and 0.1 C. The high sulfur loading S@CoS_2_‐LBLCN cathodes also show good cycling stabilities at high current rates of 0.5 and 1.0 C (Figure 4c). The S@CoS_2_‐LBLCN cathode initially delivers a capacity of 733.9 mAh g^−1^ at 0.5 C, which gradually increases to 915.6 mAh g^−1^ during activation and finally maintains at 793.5 mAh g^−1^ after 300 cycles. Even when the current rate increases to 1.0 C, a reversible capacity of 642 mAh g^−1^ can be achieved after 300 cycles. The corresponding charge/discharge profiles at the 1st, 127th, 200th, and 300th at 0.5 C are illustrated in Figure 4b. It is clearly observed that all curves deliver evident two‐plateau behavior during the discharge process. These plateau patterns remain unchanged even after 300 cycles, indicating the good electrochemical stability of the S@CoS_2_‐LBLCN cathode.

**Figure 5:**
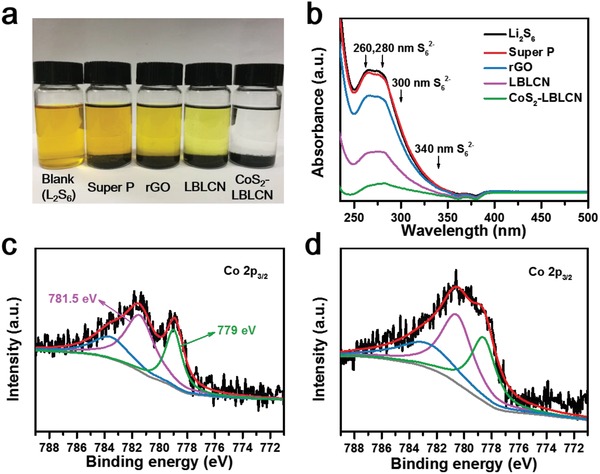
a) Digital image of polysulfides (Li_2_S_6_) adsorption test. b) UV–vis absorption spectra of Li_2_S_6_ solution after addition of Super P, rGO, LBLCN, and CoS_2_‐LBLCN. c,d) Co 2p_3/2_ XPS spectra of CoS_2_‐LBLCN before (c) and after (d) contacting with Li_2_S_6_ solution.

Static adsorption test was carried out to visually exhibit the interaction between CoS_2_‐LBLCN and polysulfides, where Li_2_S_6_ was used as the representative. Super P, rGO, LBLCN, and CoS_2_‐LBLCN were added separately into a 0.005 m Li_2_S_6_ solution. After resting for 6 h, the solution containing CoS_2_‐LBLCN appeared almost transparent while the others still showed yellow color to some extents, as shown in **Figure** **5**a. Figure 5b shows the corresponding ultraviolet–visible (UV–vis) absorption spectra of the above solutions. Broad absorbance in the 250–350 nm is observed for all samples due to the existence of S_6_^2−^ species.^33^ However, the absorbance of the solution containing CoS_2_‐LBLCN is much lower than others, which is consistent with the difference in solution color. In addition, XPS analysis was carried out to investigate the chemical interaction between CoS_2_ and polysulfides. As shown in Figure 5c,d, after Li_2_S_6_ adsorption, the characteristic Co 2p_3/2_ peaks at 781.5 and 779 eV shift toward a lower binding energy, which confirms the strong chemical interaction between the CoS_2_ and the polysulfides.^34^

**Figure 6:**
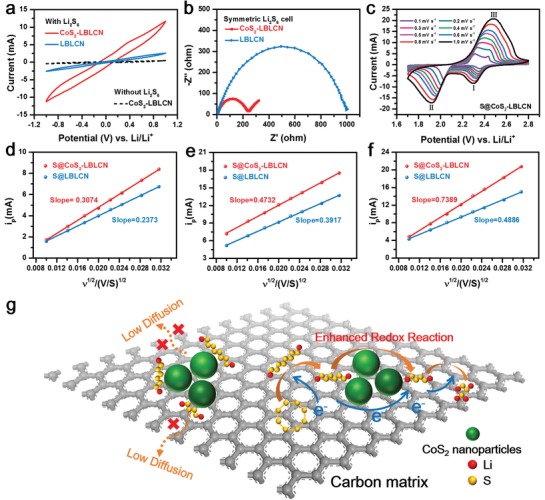
a) CV curves of the symmetric cells with and without Li_2_S_6_. b) EIS spectra of Li_2_S_6_ symmetric cells. c) CV curves of the Li–S cell with S@CoS_2_‐LBLCN cathode at different scan rates. d–f) Plots of peak current (*I*_p_) for the first cathodic reduction process (d), second cathodic reduction process (e), and anodic oxidation process (f) with the square root of the scan rate (ν^1/2^) of the Li–S cells with CoS_2_‐LBLCN and LBLCN cathodes. g) The role of CoS_2_ nanoparticles on polysulfides capture and conversion.

To investigate the effects of the CoS_2_ nanoparticles on the polysulfides redox kinetics, CV tests were conducted for symmetrical cells with and without Li_2_S_6_ over a voltage range from −1.0 to 1.0 V. As shown in **Figure** **6**a, the cell without Li_2_S_6_ shows near‐zero capacitive current density while the cells with Li_2_S_6_ exhibit some current response. More importantly, the Li_2_S_6_ symmetric cell with CoS_2_‐LBLCN electrode has the best current response, indicating CoS_2_ nanoparticles play a key role in enhancing polysulfides redox kinetics. It is also found that the Li_2_S_6_ symmetric cell with CoS_2_‐LBLCN electrode has a much smaller *R*_ct_ than the cell with LBLCN electrode (Figure 6b), confirming that CoS_2_ can effectively facilitate charge transfer and accelerate the polysulfides redox reactions. Finally, the diffusion coefficient of Li ions in the Li–S cells with CoS_2_‐LBLCN and LBLCN cathodes was characterized by CV (Figure 6c; Figure S8, Supporting Information). The diffusion coefficient of the Li ions can be described by the Randles–Sevcik equation^35^

$$I_{p}=(2.\text{69}\times {\text{10}}^{5})n^{1.5}SD^{0.5}Cv^{0.5}$$

where *I*_p_ is the peak current, *n* is the electron charge number (*n* = 2), *S* is the area of the electrode (0.95 cm^2^), *D* is the diffusion coefficient of the Li ions, *C* is the Li‐ion concentration change during reaction (0.001 mol cm^−3^), and *v* is the scan rate. Because *n*, *S*, and *C* are given data, there is a linear relationship between *I*_p_ and *v* ^0.5^, and *D* is correlated positively to the slopes of the curves (*I*_p_/*v* ^0.5^). As shown in Figure 6d–f, the slopes of the S@CoS_2_‐LBLCN cathode are higher than that of the S@LBLCN cathode in each redox reaction, which further indicates that the CoS_2_‐LBLCN endows the Li–S cells with a faster Li‐ion diffusion rate. This feature reflects that the embedded ultrafine CoS_2_ nanoparticles enable effective chemical adsorption of polysulfides and catalyze the conversion of polysulfides redox, as shown in Figure 6g.

## 3 Conclusion

A layer‐by‐layer S@CoS_2_‐LBLCN composite cathode with high electrochemical catalysis of polysulfides conversion has been developed for Li–S batteries. The LBLCN has interconnected conductive framework and hierarchical pore structure, which promotes fast transport of ions and electrons, and provides sufficient space for sulfur storage and the volumetric expansion of sulfur during cycling. In addition, the MOF‐derived ultrafine CoS_2_ nanoparticles significantly facilitate the electrochemical redox kinetics because of its strong chemical affinity to polysulfides and high electrocatalytic effect. Taking advantages of these features, Li–S batteries with S@CoS_2_‐LBLCN cathodes exhibit a long cycle life of 1000 cycles at 5.0 C with a capacity decay of 0.033% per cycle and good rate capabilities with 1180.7 and 700 mAh g^−1^ at 1.0 and 5.0 C, respectively. Designing layer‐by‐layer electrodes gives a new strategy for high rate Li–S batteries and can also be applied in other electrochemical field, such as metallic oxide/metal sulfide/metal phosphide anode, solid state electrolyte, and supercapacitor.

## 4 Experimental Section

*Synthesis of BMZIFs/GO and ZIF‐8/GO*: 1 mL of GO solution (10 mg mL^−1^) was dispersed in 20 mL methanol with ultrasonication for 30 min. Then, 100 mg of PVP was added into the GO solution and continued dispersing for 2 h. 2.0 mmol of Zn(NO_3_)_2_ · 6H_2_O and 1.0 mmol of Co(NO_3_)_2_ · 6H_2_O were dissolved in the above solution and stirred for 3 h at room temperature to form a well‐distributed solution. Subsequently, 20 mL of 2‐methylimidazole (12 mmol) methanol solution was quickly added into the above well‐distributed solution with vigorous stirring for 30 min. The as‐obtained dark purple precipitates were collected by centrifugation and washed with methanol for three times and deionized water for two times to remove impurities. Finally, the BMZIFs/GO powder was obtained by freeze‐drying for 48 h. The ZIF‐8/GO was synthesized via the same method without the addition of Co(NO_3_)_2_ · 6H_2_O.

*Synthesis of Co‐LBLCN, CoS_2_‐LBLCN, and LBLCN*: The as‐prepared BMZIFs/GO precursors were heated at 900 °C for 3 h with a heating rate of 2 °C min^−1^ under Ar flow. After cooling to room temperature naturally, the as‐obtained black powder was labeled as Co‐LBLCN. 500 mg of sublimate sulfur and 200 mg of Co‐LBLCN were separately put into two porcelain boats and placed in a tube furnace. Sublimate sulfur and Co‐LBLCN were placed in upstream and downstream of the tube furnace, respectively. The tube was heated at 400 °C for 3 h with a heating rate of 2 °C min^−1^ under Ar flow. After cooling, the black CoS_2_‐LBLCN was obtained. The LBLCN was obtained by direct carbonization of ZIF‐8/GO powder at 900 °C for 3 h.

*Synthesis of Sulfur Composite*: The S@CoS_2_‐LBLCN composite was performed via a melt‐diffusion method. The CoS_2_‐LBLCN and sulfur powder with a weight ratio of around 1:4 were ball‐milled for 30 min and sealed in a stainless steel cans, followed by heating at 155 °C for 24 h. The S@LBLCN composite was prepared by the same procedure with S@CoS_2_‐LBLCN.

*Material Characterization*: The morphology of the composites was observed using a field emission scanning electron microscope (FE‐SEM, FEI Quanta 650, USA) and HRTEM (JEOL‐2010, Japan). Nitrogen adsorption–desorption isotherms were carried out with a Quantachrome Instruments Autosorb‐IQ‐MP. XRD patterns were conducted on a diffractometer (Ultima IV‐185, Japan) with Cu Kα (λ = 0.154 nm) radiation between 10° and 80°. The mass loading of sulfur was analyzed by TGA (EXSTAR 6200, Japan) under N_2_ flow with a heating rate of 10 °C min^−1^. XPS measurements were conducted on an XPS apparatus (Thermo ESCALAB 250Xi, USA) with an Al Kα X‐ray source. The UV–vis absorption spectra were measured on a UV–vis spectrophotometer (UV‐2450, Japan) within the spectral range of 200–800 nm.

*Li_2_S_6_ Adsorption Test*: The Li_2_S_6_ solution was prepared by adding Li_2_S and S with a molar ratio of 1:5 into a 1,3‐dioxolane (DOL) and dimethoxyethane (DME) mixture (1:1 v/v) and stirred for 48 h at room temperature. About 30 mg of super P, rGO, LBLCN, and CoS_2_‐LBLCN was added into 10 mL of 5 × 10^−3^ m Li_2_S_6_ solution, respectively. Then it was rested for 6 h to evaluate the polysulfides absorption capacities of different samples.

*Assembly of Li_2_S_6_ Symmetric Cells and Measurements*: 90 wt% CoS_2_‐LBLCN or LBLCN and 10 wt% polyvinylidene fluoride (PVDF) binder were mixed in *N*‐methyl‐2‐pyrrolidone (NMP) to form a homogeneous slurry and then coated onto the Al foil. The electrodes were dried at 60 °C overnight and then cut into disks with a diameter of 11 mm. The mass loadings of both CoS_2_‐LBLCN and LBLCN electrodes were around 2.2 mg cm^−2^. Coin cells (CR2025) with two CoS_2_‐LBLCN or LBLCN electrodes as the cathode and anode were assembled in an Ar‐filled glove box. The electrolyte was 1 m bis(trifluoroethanesulfony)imide lithium (LiTFSI) and 0.5 m Li_2_S_6_ in DOL/DME mixture (1:1 v/v). The amount of electrolyte was 40 µL in each cell. CV and electrochemical impedance spectroscopy (EIS) tests were carried out on the Chenhua CHI‐660D electrochemical workstation. CV measurements were performed at a scan rate of 5 mV s^−1^ between −1 and 1 V.

*Electrochemical Measurement*: Typically, the sulfur cathodes were made by mixing 80% S@CoS_2_‐LBLCN (or S@LBLCN) and 10% super P with 10% PVDF in NMP to form a well‐distributed slurry. Then, the slurry was coated onto the Al foil and dried at 60 °C for 12 h under vacuum. The sulfur loadings were around 1.8 and 3.0 mg cm^−2^. Coin cells (CR2025) with sulfur composite as cathode, lithium metal as anode, and Celgard 2325 membrane as a separator were assembled in an Ar‐filled glove box. The electrolyte was the mixed solvent of DOL and DME (1:1, v/v) containing 1.0 m LiTFSI and 0.2 m LiNO_3_. The cycling and rate performances of the coin cells were tested using a Land CT2001A test system at different current rates with the voltage range of 1.7–2.8 V. The coin cells with S@CoS_2_‐LBLCN cathode were activated for five cycles at 0.2 C before cycling at 5.0 C. CV curves were collected on a Chenhua CHI‐660D electrochemical workstation at different scan rates from 0.1 to 2 mV s^−1^ between 1.7 and 2.8 V. The EIS was also carried out on the Chenhua CHI‐660D electrochemical workstation in the range between 10^5^ and 0.01 Hz.

## Conflict of Interest

The authors declare no conflict of interest.

## Supporting information

Supplementary
