# Supplementary material for: Boosting High‐Rate Li–S Batteries by an MOF‐Derived Catalytic Electrode with a Layer‐by‐Layer Structure
Source: Adv Sci (Weinh). 2019 Jul 15;6(16):1802362. doi: 10.1002/advs.201802362 (PMC6702624; doi:10.1002/advs.201802362)
Supplement: Supplementary file 1 — Supplementary [file ADVS-6-1802362-s001.pdf]

Copyright WILEY-VCH Verlag GmbH & Co. KGaA, 69469 Weinheim, Germany, 2019.

ADVANCED SCIENCE
Open Access

Supporting Information

for *Adv. Sci.,* DOI: 10.1002/advs.201802362

Boosting High-Rate Li–S Batteries by an MOF-Derived Catalytic Electrode with a Layer-by-Layer Structure

*Wanlong Li, Ji Qian, Teng Zhao, Yusheng Ye, Yi Xing, Yongxin Huang, Lei Wei, Nanxiang Zhang, Nan Chen, Li Li, Feng Wu, and Renjie Chen**

Copyright WILEY-VCH Verlag GmbH & Co. KGaA, 69469 Weinheim, Germany, 2019.

Supporting Information

Boosting high-rate Li-S batteries by a MOFs-derived catalytic electrode with a layered-by-layered structure

*Wanlong Li, Ji Qian, Teng Zhao, Yusheng Ye, Yi Xing, Yongxin Huang, Lei Wei, Nanxiang Zhang, Nan Chen, Li Li, Feng Wu, and Renjie Chen**

Dr. W. Li, Dr. J. Qian, Dr. T. Zhao, Dr. Y. Ye, Dr. Y. Xing, Dr. Y. Huang, Dr. L. Wei, Dr. N. Zhang, Dr. N. Chen, Prof. L. Li, Prof. F. Wu, Prof. R. Chen
Beijing Key Laboratory of Environmental Science and Engineering
School of Material Science and Engineering
Beijing Institute of Technology
Beijing 100081, PR China
E-mail: chenrj@bit.edu.cn
Prof. L. Li, Prof. F. Wu, Prof. R. Chen
Collaborative Innovation Center of Electric Vehicles in Beijing
Beijing 100081, PR China

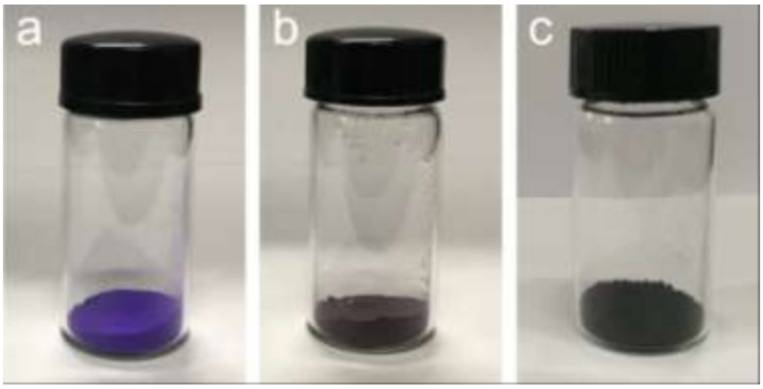

Figure S1. Digital photos of (a) BMZIFs, (b) BMZIFs/GO and (c) CoS_2-LBLCN.

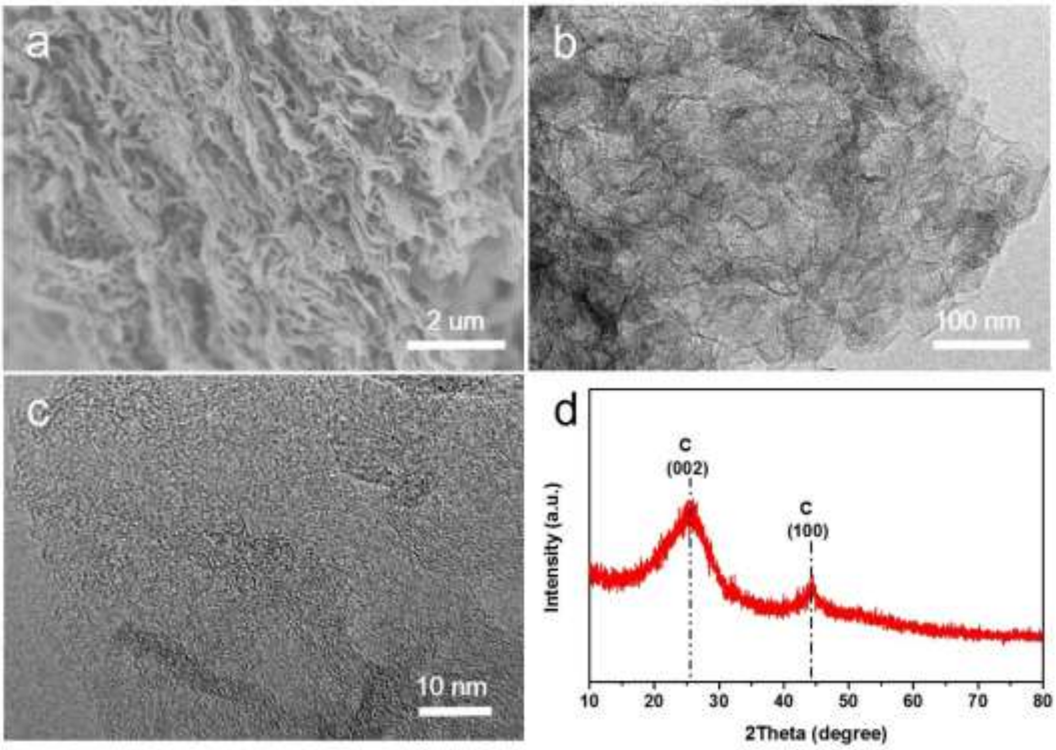

Figure S2. (a) SEM image, (b) TEM image, (c) HRTEM image and (d) XRD pattern of LBLCN.

As shown in Figure S2a, LBLCN shows a 3D layered-by-layered structure, which is similar to CoS_2-LBLCN. TEM and HRTEM images confirm the porous sheet-like structure of LBLCN without embedded CoS_2 nanoparticles. Figure S2d shows the XRD pattern of

LBLCN, the broad peak around 26° represents the interplane (002) reflection of carbon from rGO and ZIF-8-derived porous carbon.

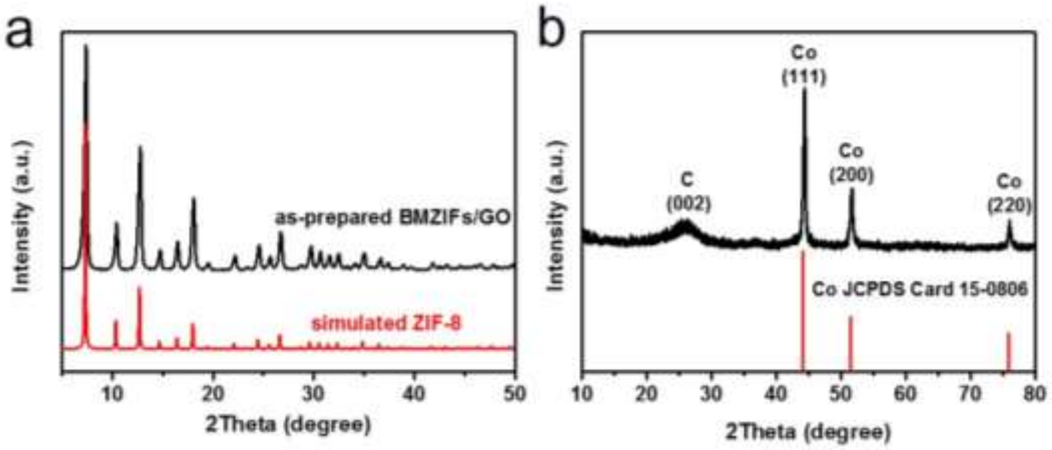

Figure S3. XRD patterns of (a) simulated ZIF-8 and as-prepared BMZIFs/GO and (b) Co-LBLCN and metallic Co (JCPDS Card No. 15-0806).

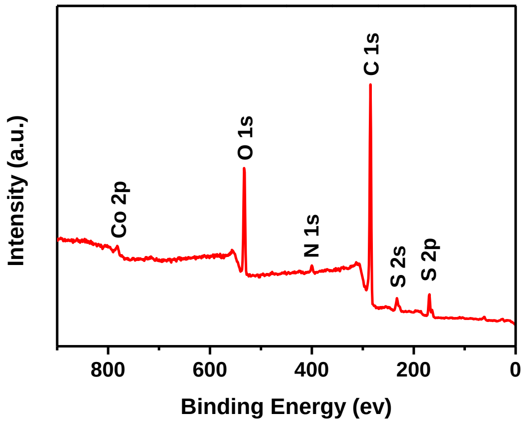

Figure S4. XPS survey spectra of CoS_2-LBLCN

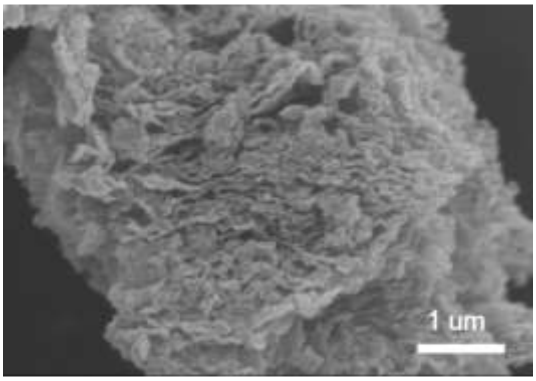

Figure S5. SEM image of S@CoS_2-LBLCN

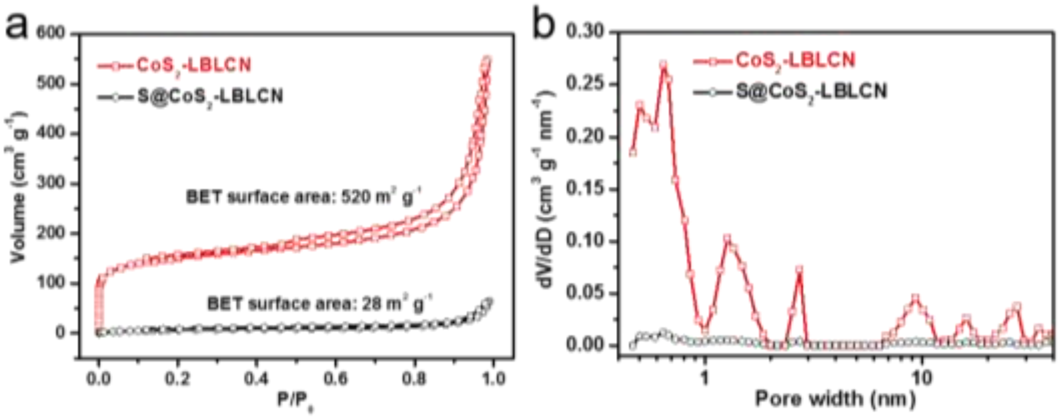

Figure S6. (a) Nitrogen adsorption-desorption isotherms and (b) pore size distributions of CoS_2-LBLCN and S@CoS_2-LBLCN.

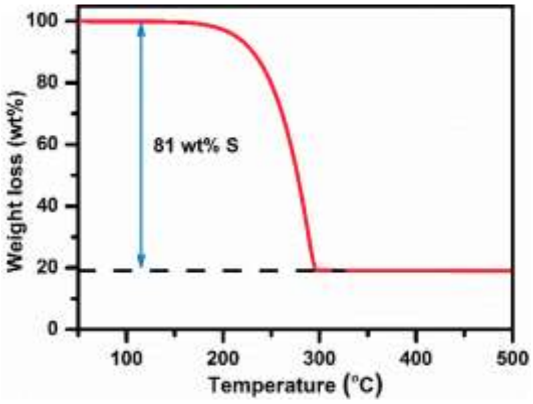

Figure S7. TGA curve of S@CoS_2-LBLCN

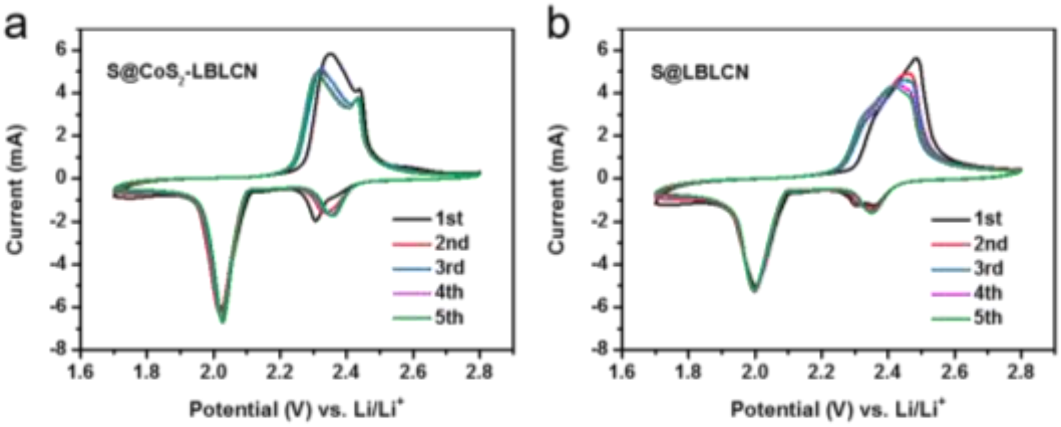

Figure S8 CV profiles of the (a) S@CoS_2-LBLCN and (b) S@LBLCN cathodes at a scan rate of 0.1 mV s^{-1}.

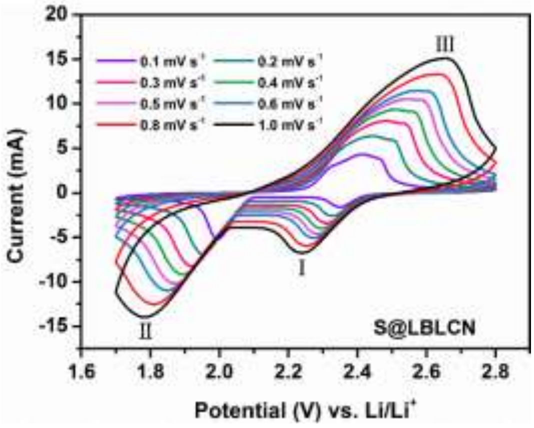

Figure S9 CV profiles of the Li-S cell with S@LBLCN cathode at different scan rates.

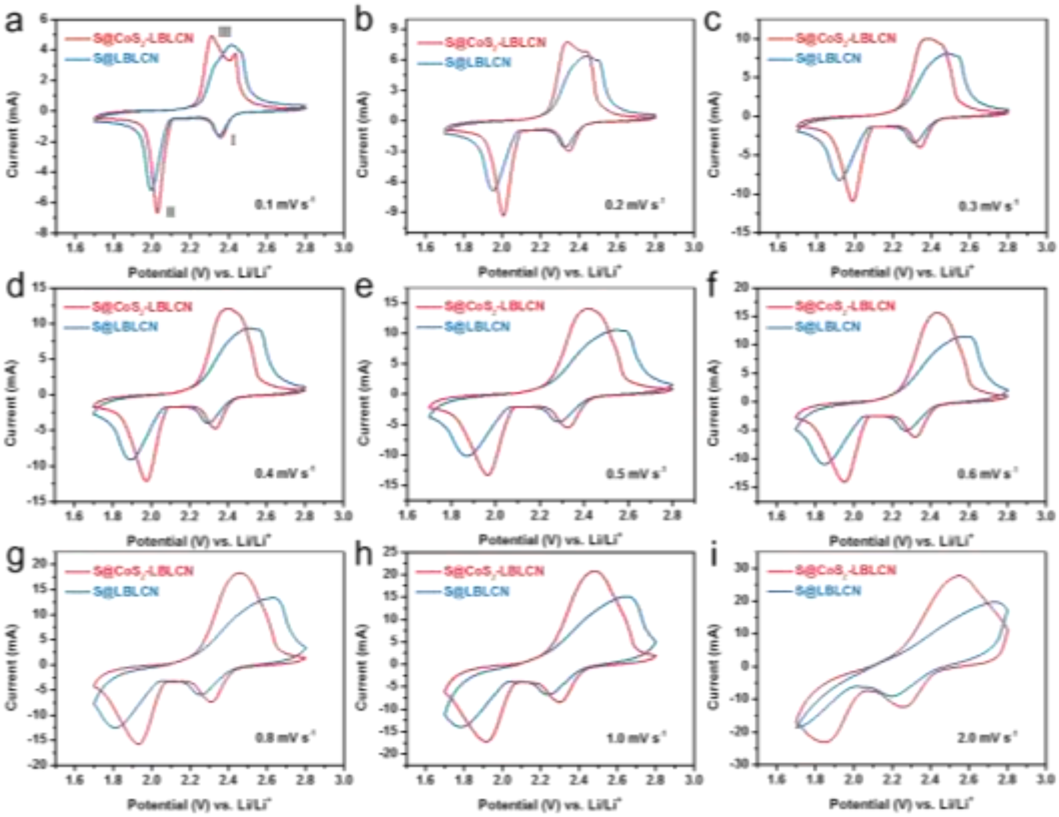

Figure S10 Comparison of CV profiles of S@CoS_2-LBLCN and S@LBLCN cathodes at different scan rates from 0.1 to 2.0 mV s^{-1}.

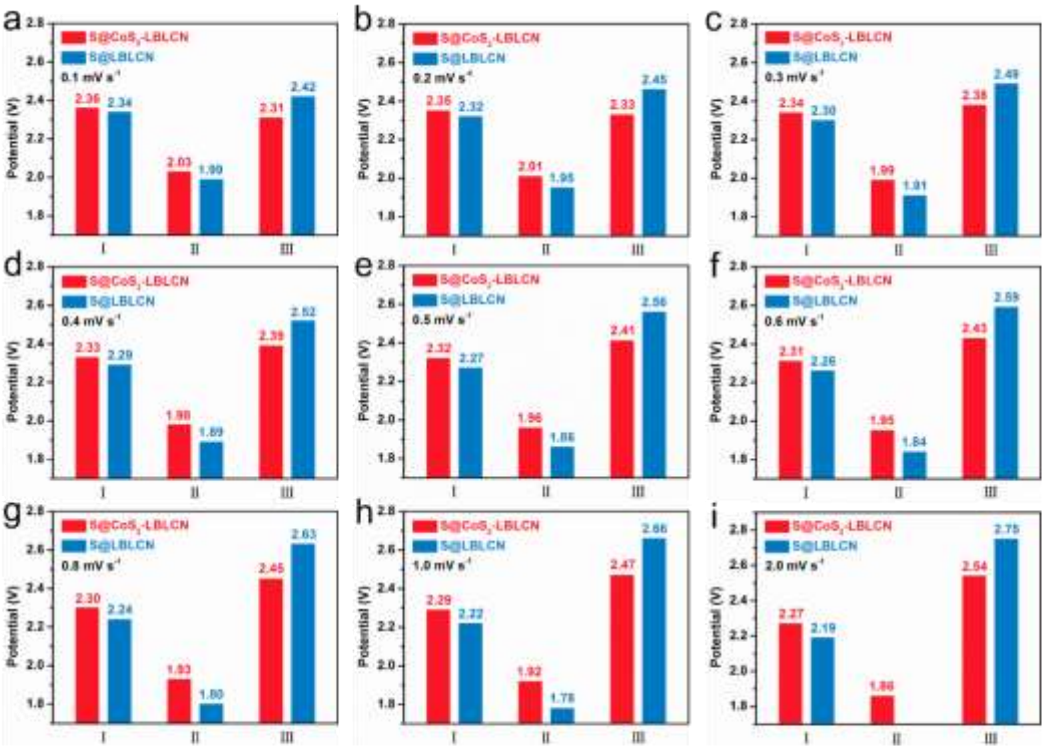

Figure S11 Comparison of corresponding peak voltages of S@CoS_2-LBLCN and S@LBLCN cathodes at different scan rates from 0.1 to 2.0 mV s^{-1}.

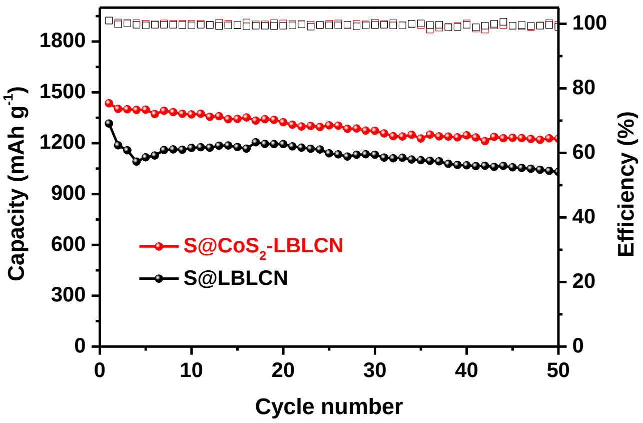

Figure S12 Cycling performances and coulombic efficiencies of S@CoS_2-LBLCN and S@LBLCN cathodes at 0.2 C.

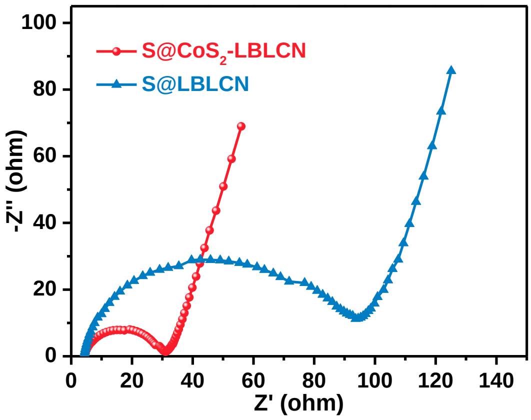

Figure S13 EIS spectra of the S@CoS_2-LBLCN and S@LBLCN cathodes before cycling.

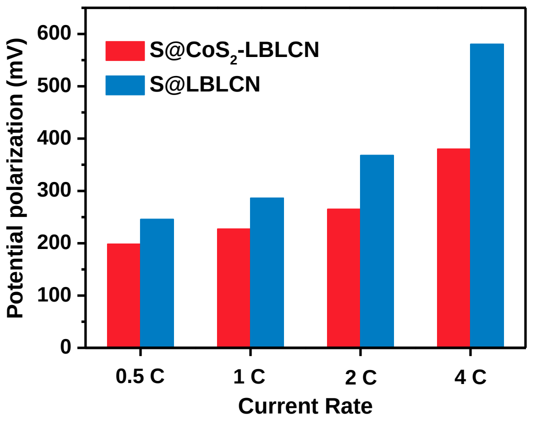

Figure S14 Comparison of the potential polarization between S@CoS_2-LBLCN and S@LBLCN cathodes at different current rates.

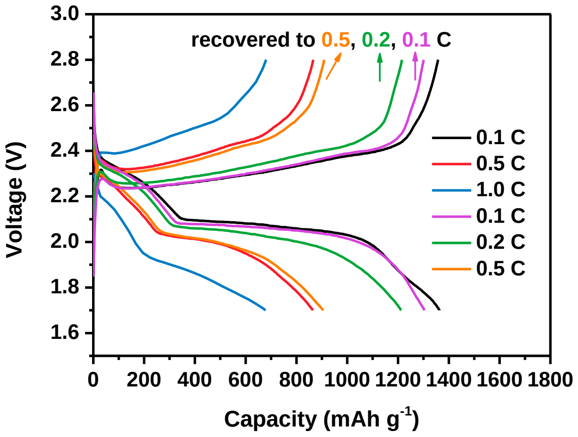

Figure S15 Charge/discharge profiles of S@CoS_2-LBLCN cathode with sulfur loading of 3 mg cm^{-2} at different rates.
